# Dento‐Skeletal Effects of the Myofunctional 8‐Appliance in Growing Patients

**DOI:** 10.1002/cre2.70368

**Published:** 2026-04-28

**Authors:** Michele d'Attilio, Valeria Pestilli, Franco Pestilli, Giulia Semerari, Giuliano Ascani, Beatrice Femminella, Antonino Peluso

**Affiliations:** ^1^ Department of Innovative Technologies in Medicine & Dentistry University “G. d'Annunzio” Chieti‐Pescara Chieti Italy; ^2^ Orthodontic Laboratory Franco Pestilli Celano Italy; ^3^ University of Chieti Chieti Italy; ^4^ Department of Maxillofacial Surgery Spirito Santo Hospital Pescara Italy; ^5^ Department of Human Sciences, Law, and Economics Telematic University Leonardo da Vinci, UNIDAV Torrevecchia Teatina Italy

**Keywords:** cephalometric analysis, dentoalveolar expansion, mixed dentition, myofunctional 8‐appliance (MF8A), orthodontic functional therapy, tongue posture

## Abstract

**Objectives:**

This study aimed to evaluate the dento‐skeletal effects of the Myofunctional 8‐Appliance (MF8A) in growing patients with transverse dentoalveolar deficits. The primary research question was whether MF8A could guide dental arch growth by supporting tongue function in patients showing transverse deficiencies.

**Materials and Methods:**

The study was conducted on 18 patients (aged 6–10 years) with mixed dentition. Patients were treated with the MF8A for at least 16 h daily and monitored monthly. Cephalometric radiographs and dental casts were obtained before treatment (T0) and after therapy (T1). Cephalometric analysis assessed skeletal and dental angular/linear changes, while 3D dental cast analysis measured arch dimensions. Statistical significance was set at *p* < 0.05.

**Results:**

Cephalometric analysis revealed no significant skeletal changes, except for an increase in the I+/ANS‐PNS angle (*p* = 0.049), suggesting improved upper incisor inclination. Dental cast analysis showed significant transverse expansion in both arches, including increased intercanine, interpremolar, and intermolar widths (*p* < 0.001). Upper and lower arch depths also increased significantly, whereas lower arch perimeter changes were not statistically significant.

**Conclusions:**

The MF8A demonstrated effectiveness in producing dentoalveolar expansion, primarily through functional re‐education of tongue posture, while exerting minimal skeletal changes.

## 1 Introduction

Function is recognized as one of the main factors in the morpho‐functional balance of the head and neck region. The balance between the forces of the labiolingual muscles and the tongue, as well as other environmental factors, is crucial in determining the shape of the dental arches and the position of the teeth (Lin et al. 2023).

The tongue plays a fundamental role in shaping the dental arches during growth and maintaining functional balance within the oral cavity.

The tongue supports the dental arches and allows for their proper development. Over time, however, malocclusions can be caused by an incorrect tongue position, such as a low posture within the oral cavity or incorrect swallowing.

During its function, the tongue exerts force on the teeth, and the dental arches develop thanks to the interaction between the tongue, teeth, and surrounding structures.

Anomalies in tongue function, such as chewing, swallowing, and resting position, are directly related to the development of malocclusions (Deshkar et al. 2024).

The forces exerted by the tongue are directly related to the transverse dimension of the dental arches (Lin et al. 2023; Deshkar et al. 2024; Deregibus et al. 2021). Bourdiol et al. (2010) demonstrated how the height of the palatal vault is directly correlated with the posture and size of the tongue, while a study by Mehra et al. (2025) has demonstrated how there is a different morphology and lingual position in patients with different skeletal classes.

These findings support the hypothesis that orofacial dysfunctions can contribute to the development of malocclusions and alterations in the position of the teeth, as well as orthodontic treatments that do not re‐educate neuromuscular function can more easily determine relapses (Ferrario et al. 2000; Van Dyck et al. 2016).

The treatment of malocclusions during the growth period involves the use of functional appliances with the aim of achieving a favorable neuromuscular balance and harmonious development of the arches and jaws.

These devices work by creating a new functional pattern that can indirectly influence the arrangement of the teeth, improve occlusion, and improve the relationship between the bone bases (Carels and Van Der Linden 1987). Muscle tone, tongue posture, and swallowing quality are key elements in promoting physiological expansion of the palate and mandibular arch (D'Onofrio 2019).

Studies by Piancino, Vallelonga et al. (2024) have demonstrated that functional devices used in the treatment of mono‐ and bilateral cross‐bite are able to correct the dental position and normalize the chewing pattern, reducing reverse chewing cycles (Piancino et al. 2017).

The aim of this study is to evaluate the dentoskeletal effects of a functional device called the myofunctional 8‐appliance (MF8A). Specifically, we intend to analyze the device's ability to promote dental arch remodeling, supporting and enhancing the functional action of the tongue in cases where the latter has been insufficient to properly guide the development and shape of the arches.

## 2 Materials and Methods

### 2.1 Myofunctional 8‐appliance (MF8A)

**Figure 1:**
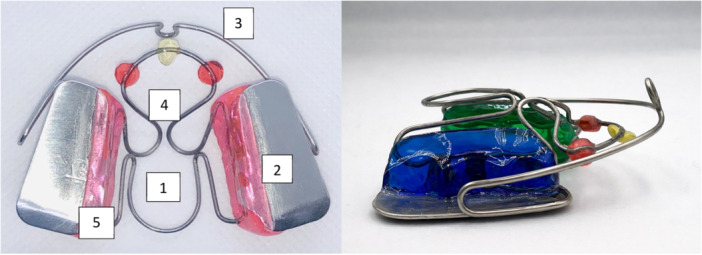
Occlusal and lateral view of the appliance. 1‐Figure 8 spring; 2‐metallic bite; 3‐vestibular arc; 4‐lingual stimuli; 5‐acrylic resin.

The MF8A (Figure 1) consists of the following components:

– Figure 8 spring: The figure‐8‐shaped spring is located at the center of the device and can be divided into anterior and posterior sections. The spring is made of titanium‐molybdenum alloy (TMA) and is connected to the resin bodies located on the sides.
  The spring is the main component of the device, as it allows each swallowing action to exert both a transverse and sagittal expansion force thanks to the thrust of the tongue.
– Metallic bite: Posterior metal bites (D.M. CEOSA, Madrid, Spain) are made from the distal canine to the last tooth in the arch. They must be positioned parallel to the occlusal plane and are embedded in the resin shields.
– Lingual stimuli: Located in the anterior portion of the figure‐8 spring, there are three lingual stimuli: the central one, shaped like a retro incisive papilla, positioned above the palatal spot, and the two lateral hemispherical ones positioned lateral to the first palatal rugae. The lingual stimuli can also be positioned individually depending on the patient's needs.
– Resin shields: Located in the upper posterior lateral regions, the resin shields extend from the distal canine to the distal end of the last tooth in the arch and are in contact with the alveolar processes. From the neck of the teeth, the resin shields extend apically for 5–6 mm. The shields connect the wires that make up the figure‐8 spring and are concave in shape to guide the tongue towards the spring during swallowing. ‐ Vestibular arch: The vestibular arch must be 2 mm away from the vestibular surface of the anterior teeth and has a central activation loop to allow subsequent activations. It can be made of steel or cobalt‐chromium with a 1 mm diameter wire.

### 2.2 Patient Recruitment and Treatment

The study was conducted at the unit of orthodontic, Department of Innovative Technologies in Medicine and Dentistry, University of Chieti, Italy.

Patients underwent an orthodontic examination and were evaluated by a single operator (M.D.A.).

The inclusion criteria were as follows:

– Transverse dentoalveolar deficit
– Negative torque of the posterior lateral segments
– Mixed dentition
– No previous orthodontic treatment

The exclusion criteria were:

– No‐compliance patients
– Hereditary or congenital disease

The transverse dentoalveolar deficit was evaluated clinically and confirmed through visual assessment of the WALA ridge on intraoral frontal photographs, according to the method described by Ramón et al. (2022). The WALA ridge assessment served to verify the absence of a skeletal basal transverse discrepancy, thereby confirming the dentoalveolar nature of the transverse deficit. Negative torque of the posterior segments was assessed through clinical examination.

The enrolled patients underwent orthodontic treatment with the MF8A device. Upon delivery of the device, patients were instructed to use it for a minimum of 16 h per day. Patients underwent monthly checks. Teleradiographs and dental casts were obtained before the start of therapy (T0) and at the end of the orthopedic‐functional treatment (T1).

All study participants signed an informed consent; the study was conducted in observance of the Helsinki and Good Clinical Practice Guidelines. The STROBE statement has been followed in the reporting of this study (Vandenbroucke et al. 2007).

### 2.3 Cephalometric Analysis

Cephalometric radiographs were performed at the radiology unit of the Dental Clinic of the G. d'Annunzio University. Patients were informed of the examination they would undergo, had any metal objects removed, and their hairs, if necessary, were tied back to not interfere with the radiological field.

The patient was positioned upright between the lateral auricular supports of the craniostat with the Frankfurt plane parallel to the floor, teeth in habitual occlusion, lips closed, and tongue on the palate.

Before leaving the room, the operator checked the patient's correct position, and then the cephalometric radiograph was performed.

Cephalometric radiographs were performed at T0 and T1.

The cephalometric radiographs were subsequently analyzed with OrisCeph3 software (OrisLine; Elite Computer Italia S.r.l., Milan, Italy) using the parameters shown in Table 1.

**Table 1:** Variables measured for the cephalometric analysis.

Index	Normal value and SD	Description
SNA	82 ± 2	Angle formed between points S, N e A
SNB	80 ± 2	Angle formed between points S, N e B
ANB	2 ± 2	Angle formed between points A, N e B
Wits appraisal	0 ± 2	Distance between points A and B reported orthogonally on the Ricketts occlusal plane
A – McNamara	Mixed dentition: −1 Permanent dentition: 0	Distance from point A to the McNamara line (line passing through point N perpendicular to FH)
Pog – McNamara	9 years: Male −6 Female −8 12 years: Male −5 Female −5 15 years: Male −2 Female −2 16 years: Male 0 Female 0 18 years: Male 0 Female 0	Distance of point Pog from the McNamara line (line passing through point N perpendicular to the Frankfurt line)
SN^GoGn	32 ± 5	Angle between the cranial base (SN) and the geometric mandibular plane (GoGn)
FMA	26 ± 5	Angle between the Frankfort plane and the anatomical mandibular plane (GoMe)
MM	27 ± 5	Angle between the bispinal line (ANS‐PNS) and the anatomical mandibular plane (GoMe)
I+/SN	103 ± 2	Angle between the upper incisor and the plane of the cranial base
I+/FH	Hypodivergent: 113 ± 1 Normodivergent: 110 ± 1 Hyperdivergent: 107 ± 1	Angle between the upper incisor and the Frankfort plane
I+/ANS‐PNS	Hypodivergent: 113 ± 2 Normodivergent: 110 ± 2 Hyperdivergent: 107 ± 2	Angle between the upper incisor and the bispinal plane
I+/APog	3.5 ± 2	Distance between the upper incisor and the line passing through points A and Pog
I‐/APog	2 ± 2	Distance between the lower incisor and the straight line passing through points A and Pog
IMPA	Hypodivergent: 93 ± 3 Normodivergent: 90 ± 3 Hyperdivergent: 87 ± 3	Angle between the lower incisor and the anatomical mandibular plane
I+/I−	130 ± 5	Angle between the axis of the upper incisor and the axis of the lower incisor
Sellar angle	122 ± 5	Angle formed between points N, S e Ar
Articular angle	143 ± 6	Angle formed between points S, Ar e Go
Gonial angle	120 ± 5	Angle formed between points Ar, Go e Me
Superior gonial angle	50 ± 2	Superior angle formed from the total goniac angle by drawing a straight line passing through Go and N
Inferior gonial angle	70 ± 3	Lower angle formed from the total goniac angle by drawing a straight line passing through Go and N

### 2.4 Dental Cast Analysis

Dental cast obtained from the analogic impressions was scanned and digitized with the Open Technologies laboratory scanner (Open Tech 3D s.r.l., Rezzato, Italy).

The digital dental cast obtained were then analyzed using Maestro 3D Dental Studio V5 software (AGE Solutions s.r.l., Pontedera, Italy) to measure:

– the transverse diameters at the upper and lower intermolar level
– the transverse diameters at the upper and lower interpremolar level
– the transverse diameters at the upper and lower intercanine level
– the arch perimeter
– the depth of the palate and sublingual area in both arches

**Figure 2:**
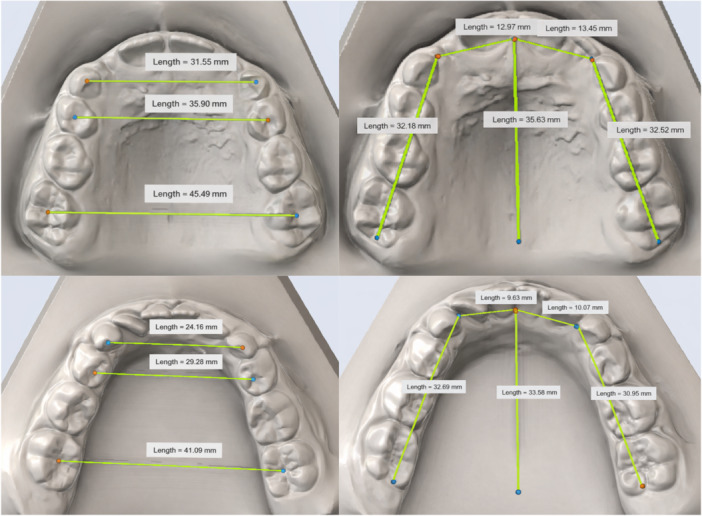
Dental cast measurements.

All points measured on the digital models are listed and explained in detail in Table 2 and shown in Figure 2.

**Table 2:** Variables measured for the dental cast analysis.

U3L‐U3R	Distance between the cusp of the left canine and the cusp of the right canine, in the upper arch
U4L‐U4R	Distance between the central fossa of the first premolar/first deciduous molar on the left and the central fossa of the first premolar/first deciduous molar on the right, in the upper arch
U6L‐U6R	Distance between the central fossa of the left first molar and the central fossa of the right first molar, in the upper arch
L3L‐L3R	Distance between the cusp of the left canine and the cusp of the right canine, in the lower arch
L4L‐L4R	Distance between the central fossa of the first premolar/first deciduous molar on the left and the central fossa of the first premolar/first deciduous molar on the right, in the lower arch
L6L‐L6R	Distance between the central fossa of the left first molar and the central fossa of the right first molar, in the lower arch
UAP (upper arch perimeter)	Perimeter of the upper arch. Calculated from the distal first molar to the mesial canine and from the mesial canine to the mesial central incisor on both the right and left sides.
LAP (lower arch perimeter)	Perimeter of the lower arch. Calculated from the distal first molar to the mesial canine and from the mesial canine to the mesial central incisor on both the right and left sides.
UAD (upper arch depth)	Depth of the upper arch. Calculated from the interincisal contact point to a line tangent to the most distal points of the first molar crowns.
LAD (lower arch depth)	Depth of the lower arch. Calculated from the interincisal contact point to a line tangent to the most distal points of the first molar crowns.

### 2.5 Statistical Analysis

Statistical analysis was performed for both cephalometric variables and those obtained from digital model analysis.

Descriptive statistics are presented, and comparisons were made between the variables at T0 and those at T1.

Normal distribution was assessed using the Shapiro–Wilk test, and depending on the variable's distribution type (normally distributed or not), a parametric test (Student's *t*‐test) or a nonparametric test (Mann–Whitney test) was used, respectively.

The level of statistical significance was set at *p*‐value < 0.05, and the software used was Jamovi version 2.6.44.

## 3 Results

Eighteen patients (9 males and 9 females) with a mean age at T0 of 7.8 ± 1.3 years and at T1 of 9.0 ± 1.4 years. The mean treatment duration was 14 ± 2 months. Statistical analysis compared cephalometric values obtained at time T0 (pre‐treatment) with those at time T1 (post‐treatment), with a significance level set at *p* < 0.05.

### 3.1 Results of Cephalometric Analysis

Cephalometric data analysis showed that, when comparing T0 (pre‐treatment) and T1 (post‐treatment), most angular and linear variables did not show statistically significant differences. The only parameter that showed a significant change was the I+/ANS‐PNS angle, which increased significantly from 110.3 ± 8.66° to 114.16 ± 3.95° (*p* = 0.05). This result suggests a greater inclination of the upper incisors relative to the bispinal plane, potentially related to a change in tongue function or interaction with the palatal spring of the MF8A device.

All other cephalometric parameters remained essentially stable. The main sagittal angles, such as SNA (from 84.8° to 83.3°; *p* = 0.07), SNB (from 79.9° to 79.3°; *p* = 0.34), and ANB (from 4.86° to 3.94°; *p* = 0.11), did not undergo significant changes, indicating that the anteroposterior relationship between the skeletal bases remained essentially unchanged over the treatment period. However, a slight tendency toward a reduction in ANB was observed, which could indicate a partial improvement in the maxillomandibular sagittal relationship.

Vertical growth parameters, such as the SN^GoGn angles (from 29.17° to 30.06°; *p* = 0.09), FMA and MM, also did not show statistically significant changes. Similarly, no significant changes were observed in incisor inclinations (I+/SN, I+/FH, IMPA) or interincisive relationships (I+/I−), which remained overall stable.

Finally, craniofacial angles (sellar, articular, superior, inferior, and total goniac) did not show significant differences between T0 and T1.

Overall, these results confirm that MF8A exerts a predominantly dentoalveolar action, with limited effects on the facial skeleton. Complete cephalometric analysis data are reported in Table 3.

**Table 3:** Statistical results of cephalometric analysis. Bold value indicate statistically significant differences (*p* < 0.05)

	T0 *N* = 18			T1 *N* = 18			
Cephalometric parameters	Mean	SD	IC 95%	Mean	SD	IC 95%	*p*‐value
SNA	84.8	5.74	81.9; 87.6	83.3	4.55	81; 85.5	0.07
SNB	79.9	4.30	77.7; 82.0	79.3	3.87	77.4; 81.3	0.34
ANB	4.86	2.62	3.56; 6.16	3.94	2.32	2.79; 5.10	0.11
Wits appraisal	2.81	2.75	1.44; 4.17	1.76	2.64	0.44; 3.08	0.2
A – McNamara	2.33	6.22	−0.76; 5.42	1.97	4.26	−0.15; 4.09	0.89
Pog – McNamara	−2.93	8.36	−7.09; 1.23	−1.18	7.03	−4.68; 2.31	0.3
SN^GoGn	29.17	4.40	26.9; 31.36	30.06	4.36	27.8; 32.22	0.09
FMA	21.7	3.10	20.2; 23.3	21.4	4.71	19; 23.7	0.71
MM	22.1	3.77	20.3; 24	21	3.95	19.1; 23	0.24
I+/SN	103.3	8.8	107.7; 106	105.1	3.69	103.2; 106.9	0.33
I+/FH	110.7	8.6	106.5; 115.06	113.8	5.33	111.1; 116.4	0.11
I+/ANS‐PNS	110.3	8.66	106; 114.6	114.16	3.95	112.1; 116.1	**0.049**
I+/APog	5.16	4.15	3.09; 4.21	5.62	2.82	4.21; 7.03	0.21
I−/APog	1.13	2.14	0.06; 2.20	1.66	2.12	0.6; 2.71	0.28
IMPA	95.89	7.6	92.11; 99.68	97.58	7.67	93.7; 101.39	0.20
I+/I−	131.57	15.23	123.99; 139.14	127.23	10.53	121.99; 132.47	0.13
Sellar angle	121.86	4.44	119.65; 124.07	122.13	4.74	119.77; 124.48	0.73
Articular angle	139.06	5.13	136.5; 141.61	140.4	5.48	137.67; 143.13	0.15
Gonial angle	128.27	5.27	125.64; 124.4	127.51	6.12	124.46; 130.55	0.49
Superior gonial angle	57.7	3.48	56.05; 59.52	56.99	4.17	54.9; 59.06	0.22
Inferior gonial angle	70.5	3.17	68.9; 72.08	70.5	2.96	69.02; 71.97	0.47

### 3.2 Results of Study Cast Measurement

The results of the statistical analysis conducted on the digital models, detailed in Table 4, show statistically significant changes in almost all the parameters considered, demonstrating the effectiveness of the MF8A device in inducing morphological changes in the dental arches.

**Table 4:** Study cast analysis results. Bold value indicate statistically significant differences (*p* < 0.05)

	T0 *N* = 18			T1 *N* = 18			
Variables	Mean	SD	IC 95%	Mean	SD	IC 95%	*p*‐value
U3L‐U3R	30	3.22	28.4; 31.6	32.2	3.45	30.5; 34	**< 0.001**
U4L‐U4R	34.2	2.85	32.8; 35.6	36.4	3.14	34.8; 38	**< 0.001**
U6L‐U6R	44.7	4.18	42.7; 46.7	46.9	4.18	44.8; 48.9	**< 0.001**
L3L‐L3R	24.8	2.23	23.7; 25.9	26.8	2.37	25.6; 28	**< 0.001**
L4L‐L4R	30.2	2.14	29.1; 31.3	32.1	3.05	30.6; 33.6	**< 0.001**
L6L‐L6R	40.5	3.21	38.9; 42.1	42.1	3.31	40.4; 43.7	**< 0.001**
UAP (upper arch perimeter)	91.1	6.52	87.8; 94.3	93.4	7.75	89.6; 97.3	**0.009**
LAP (lower arch perimeter)	86.8	3.87	84.9; 88.8	88.7	4.98	86.2; 91.1	0.079
UAD (upper arch depth)	38.9	3.26	37.3; 40.5	40.6	3.53	38.8; 42.3	**< 0.001**
LAD (lower arch depth)	36.2	3.09	34.6; 37.7	38.2	2.76	36.8; 39.6	**0.004**

In the upper arch, a significant increase in all transverse diameters was observed. In particular, the intercanine distance (U3L–U3R) increased from 30 ± 3.22 mm to 32.2 ± 3.45 mm (*p* < 0.001), the interpremolar distance (U4L–U4R) from 34.2 ± 2.85 mm to 36.4 ± 3.14 mm (*p* < 0.001), and the intermolar distance (U6L–U6R) from 44.7 ± 4.18 mm to 46.9 ± 4.18 mm (*p* < 0.001). The upper arch perimeter (UAP) also showed a significant increase, going from 91.1 ± 6.52 mm to 93.4 ± 7.75 mm (*p* = 0.009). Similarly, the upper arch depth (UAD) increased significantly from 38.9 ± 3.26 mm to 40.6 ± 3.53 mm (*p* < 0.001).

Regarding the lower arch, a significant transverse widening was also recorded. The intercanine distance (L3L–L3R) increased from 24.8 ± 2.23 mm to 26.8 ± 2.37 mm (*p* < 0.001), while the interpremolar distance (L4L–L4R) increased from 30.2 ± 2.14 mm to 32.1 ± 3.05 mm (*p* < 0.001). A statistically significant increase was also observed for the lower intermolar distance (L6L–L6R), which increased from 40.5 ± 3.21 mm to 42.1 ± 3.31 mm (*p* < 0.001). Similar to the upper arch, the lower arch depth (LAD) also increased significantly, from 36.2 ± 3.09 mm to 38.2 ± 2.76 mm (*p* = 0.004).

The only exception was the lower arch perimeter (LAP), which, despite increasing from 86.8 ± 3.87 mm to 88.7 ± 4.98 mm, did not reach the threshold of statistical significance (*p* = 0.079).

Overall, these results indicate that the use of the MF8A resulted in significant arch expansion both transversally and sagittally, especially at the maxillary level, confirming the active action of the device in promoting functionally guided dento‐alveolar modifications.

## 4 Discussion

The results obtained in this study confirm the effectiveness of the MF8A device in inducing significant dentoalveolar changes in the transverse and sagittal arches.

Specifically, model analysis revealed a significant increase in intercanine, interpremolar, and intermolar distances in both the upper and lower arches, as well as an increase in arch depth.

These results suggest a direct effect of the device on arch morphology, consistent with the clinical goal of correcting transverse discrepancies in mixed dentition.

These results can be explained by the dual mechanism of action of the MF8A. During each swallowing act, the tongue activates the figure‐8 TMA spring, which allows the force exerted by the tongue to be transferred to the alveolar processes of the upper arch, promoting physiologically guided transverse expansion.

Additionally, the lingual stimuli positioned at the palatal spot guide the tongue toward a more physiological resting position against the palate.

Current literature supports the use of functional appliances to correct malocclusions and associated chewing dysfunctions, such as chewing pattern and neuromuscular balance (Piancino et al. 2017; Tortarolo et al. 2023). and that these results remain stable in the long term (Piancino et al. 2016).

The transverse widening observed at the mandibular level is due to a functional adaptation mechanism in response to maxillary expansion. The literature recognizes that maxillary expansion can induce an adaptive response of the mandibular arch (Laganà et al. 2023; Aboalnaga et al. 2021). More specifically, the mandibular transverse changes observed are attributable to a dentoalveolar uprighting of the posterior teeth. The re‐establishment of a correct maxillary transverse dimension may lead to a spontaneous buccal uprighting of the lower molars and premolars, thereby increasing the measured interarch widths at the crown level.

Cephalometric data show that the only statistically significant variable is I+/ANS‐PNS, suggesting a change in the inclination of the upper incisors, which is also most likely secondary to an improvement in the resting position of the tongue, which becomes physiological.

All other skeletal parameters remain unchanged, confirming that the action of the MF8A is limited to the dentoalveolar level.

The absence of significant changes in vertical cephalometric parameters such as SN^GoGn and ArGoMe confirms the absence of unfavorable mandibular posterior rotation during treatment.

This finding is consistent with other devices present in the literature, such as the Function Generating Bite (FGB) (Tortarolo et al. 2023) and the Anterior Bite Plane Functional Appliance (ABPFA) (Ciavarella et al. 2017).

In particular, these results demonstrate how the MF8A allows for a transverse expansion of the upper arch without determining a mandibular post‐rotation, unlike expansion with a rapid palatal expander (Chhutani et al. 2020; Conroy‐Piskai et al. 2016).

This approach represents a clinical advantage in the treatment of hyperdivergent patients, in whom maintaining the vertical dimension is crucial.

Finally, the stability of the results obtained with myofunctional devices is a crucial element. Piancino, Tortarolo et al. (2024) have documented that the expansion obtained with FGB remains stable even after several years, with a mean follow‐up of 3.7 ± 1.6 years. Although our study did not include a follow‐up, these data suggest that properly guided functional action can lead to long‐lasting results, especially when combined with effective neuromuscular re‐education.

The lack of a control group represents a methodological limitation. However, the magnitude of the transverse changes observed in the present study exceeds the increments attributable to normal growth during the mixed dentition period. According to the longitudinal data reported by Bishara et al. (1997), intercanine and intermolar widths increase significantly between ages 3 and 13, but the mandibular intercanine width is essentially established by age 8, with subsequent changes being minimal or slightly negative. Notably, in the Bishara et al. sample, maxillary intermolar width increased by only 2.4 mm in males and 2.0 mm in females over a 5‐year period from age 8 to 13, corresponding to less than 0.5 mm per year. Sangwan et al. (2011) reported that over a 3‐year observation period from primary (4–5 years) to early mixed dentition (7–8 years), the physiological increase in intercanine width was 3.93 mm, whereas intermolar width increased by only 1.49 mm. Thilander (2009), in a longitudinal study of subjects with normal occlusion from 5 to 31 years of age, confirmed that most transverse arch dimensional changes occur during the early mixed dentition, with minimal increments observed after the eruption of permanent canines and first premolars. Furthermore, Louly et al. (2011) demonstrated that in the mixed dentition period from 9 to 12 years, transverse arch width changes were non‐significant, with maxillary intercanine width increasing by only 1.2 mm (from 26.2 to 27.4 mm, *p* = 0.498) and maxillary intermolar width by 1.7 mm (from 46.7 to 48.4 mm, *p* = 0.459) over the entire 3‐year period. In the present study, the MF8A produced in a mean treatment time of approximately 14 months an upper intercanine expansion of 2.2 mm, upper interpremolar expansion of 2.2 mm, and upper intermolar expansion of 2.2 mm. These increments, particularly at the premolar and molar levels, substantially exceed the expected physiological growth for an equivalent observation period (Bishara et al. 1997; Thilander 2009; Louly et al. 2011). Similarly, the mandibular arch showed transverse increments (intercanine: 2.0 mm; interpremolar: 1.9 mm; intermolar: 1.6 mm) that cannot be explained by growth alone, since mandibular intercanine width is largely established by age 8 (Bishara et al. 1997) and intermolar increases during this developmental period are negligible (Sangwan et al. 2011; Louly et al. 2011). These comparisons with normative growth data strongly suggest that the dentoalveolar changes observed are primarily attributable to the therapeutic effect of the MF8A device rather than to physiological growth. Future studies should include a larger sample size and include comparisons with groups treated with other functional devices, or with no treatment, to strengthen the validity of the results. Although the sample size is relatively small (*N* = 18), a post‐hoc power analysis revealed that the statistical power exceeded 0.99 for all primary transverse variables (Cohen's *d* ranging from 1.22 to 2.31), confirming that the sample was adequate to detect the observed treatment effects. Furthermore, long‐term follow‐up will be essential to assess the stability of the changes achieved and their relationship with the patient's neuromuscular and skeletal development.

## 5 Conclusion

In conclusion, the MF8A is a device capable of promoting dentoalveolar expansion of the arches by leveraging lingual muscle function. Indeed, the ability to re‐educate tongue function produces secondary effects at the dentoalveolar level, manifesting through the expansion and harmonization of both arches.

The stability of vertical cephalometric parameters indicates that the MF8A does not induce unfavorable clockwise mandibular rotation. This vertical control represents a clinically relevant advantage, particularly in the management of hyperdivergent patients.

From the clinical study conducted, we consider the MF8A a promising functional device capable of reshaping the dental arches in cases where tongue function alone has been insufficient.

## Author Contributions

Conceptualization: Michele d'Attilio and Franco Pestilli. Data curation: Valeria Pestilli and Antonino Peluso. Formal analysis: Antonino Peluso. Investigation: Michele d'Attilio, Valeria Pestilli, and Antonino Peluso. Writing – original draft: Antonino Peluso, Beatrice Femminella, Giuliano Ascani, and Giulia Semerari. Writing – review and editing: Antonino Peluso, Beatrice Femminella, Giuliano Ascani, and Giulia Semerari.

## Funding

The authors have nothing to report.

## Consent

Informed consent was obtained from the legal guardian of the patient involved.

## Conflicts of Interest

The authors declare no conflicts of interest.

## Data Availability

The data that support the findings of this study are available on request from the corresponding author. The data are not publicly available due to privacy or ethical restrictions.
